# Sustainable remediation of chromium-contaminated soils: boosting radish growth with deashed biochar and strigolactone

**DOI:** 10.1186/s12870-024-04791-5

**Published:** 2024-02-16

**Authors:** Uzma Younis, Subhan Danish, Rahul Datta, Tahani Awad Alahmadi, Mohammad Javed Ansari

**Affiliations:** 1 Botany Department, The Islamia University of Bahawalpur, Sub Campus Rahim Yar Khan, Rahim Yar Khan, Punjab Pakistan; 2 Department of Soil Science, Faculty of Agricultural Sciences and Technology, Bahauddin Zakariya University, Multan, Punjab Pakistan; 3 Department of Geology and Pedology, Faculty of Forestry and Wood Technology, Mendel University in Brno, Zemedelska 1, Brno, 61300 Czech Republic; 4 Department of Pediatrics, College of Medicine and King Khalid University Hospital, King Saud University, Medical City, PO Box-2925, 11461 Riyadh, Saudi Arabia; 5 Department of Botany, Hindu College Moradabad (Mahatma Jyotiba Phule Rohilkhand University Bareilly), Moradabad, India

**Keywords:** Antioxidant activity, Chlorophyll content, Chromium, Deashed biochar, Strigolactone

## Abstract

Chromium (Cr) stress significantly hinders crop production by disrupting nutrient uptake, impairing plant growth, and contaminating soil, posing a substantial threat to agricultural sustainability. The use of deashed biochar (DAB) and strigolactone can be an effective solution to mitigate this issue. Deashed biochar enhances crop production by improving soil structure, water retention, and nutrient availability while mitigating the bioavailability of toxic substances. Strigolactone boosts plant growth by stimulating root growth, branching, shoot formation, and overall plant physiology. Nevertheless, the scientific rationale behind their collective use as an amendment to counter Cr stress remains to be substantiated. Therefore, in this study, a blend of DAB and strigolactone was employed as additives in radish cultivation, both in the absence of Cr stress and under the influence of 200Cr stress. Four treatments, i.e., 0, 20µM Strigolactone, DAB, and 20µM Strigolactone + DAB, were applied in four replications following a completely randomized design. Results demonstrate that 20µM Strigolactone + DAB produced significant improvement in radish shoot length (27.29%), root length (45.60%), plant fresh weight (33.25%), and plant dry weight (78.91%), compared to the control under Cr stress. Significant enrichment in radish chlorophyll a (20.41%), chlorophyll b (58.53%), and total chlorophyll (31.54%) over the control under Cr stress, prove the efficacy of 20µM Strigolactone + DAB treatment. In conclusion, 20µM Strigolactone + DAB is the recommended amendment for mitigating Cr stress in radish. Farmers should consider using Strigolactone + DAB amendments to combat Cr stress and enhance radish growth, contributing to a more resilient agricultural ecosystem.

**Supplementary Information:**

The online version contains supplementary material available at 10.1186/s12870-024-04791-5.

## Introduction

The rapid growth of industrialization and urbanization has led to increased environmental contamination, particularly by heavy metals [1–3]. Heavy metal pollution is distinctive because these pollutants have limited mobility and do not naturally degrade, resulting in their accumulation in soil [4–6]. This poses ongoing threats to the environment and human health, leading to soil degradation, reduced crop yields, and reduced crop quality, all detrimental to human well-being [7]. The occurrence of heavy metal contamination in global soil and water sources underscores the urgent need for effective mitigation strategies. Chromium (Cr), found in trace amounts in the atmosphere, water, and soil, primarily affects plant content through soil Cr levels, with the highest concentrations in green plant parts [8]. Cr exists in two stable valence states, Cr(III) and Cr(VI), with the latter being more mobile, bioavailable, and considerably more toxic. Cr's environmental circumstances involve dynamic processes influenced by redox reactions, precipitation dissolution, and adsorption. Certain Cr forms in soil, especially the water-soluble and ion-exchange states, are highly mobile and bioavailable [9]. While Cr is essential for plant growth, excessive concentrations can hinder development, affecting nutrient uptake and photosynthesis, causing lipid peroxidation, and altering antioxidant activity. Excess Cr damages plant roots, reduces enzyme activity, and harms plant tissues. Soil microbes are also adversely impacted by Cr, reducing their numbers and enzyme activity. In humans, Cr accumulation can result in health problems, including anemia, neuritis, and even lung cancer or death. Addressing Cr pollution in the soil is paramount to safeguard ecosystems, crops, and human health [10].

While various approaches, such as phytoremediation and chemical stabilization, have been employed to address heavy metal contamination, these methods have limitations regarding effectiveness, cost, and sustainability. There is a need for innovative and eco-friendly solutions to combat the adverse effects of heavy metal contamination [11, 12]. Activated carbon, commonly known as biochar, stands out as a prominent solution to combat prevalent challenges in agriculture [11, 13–15]. Derived from the pyrolysis of organic matter like agricultural waste or wood chips, biochar, rich in carbon content, serves multiple purposes [12, 16, 17]. It proves beneficial by enriching soil fertility, bolstering nutrient retention, and fostering microbial activity [18–20]. Its advantages extend to augmenting soil's water-holding capacity, amplifying nutrient accessibility for plants, and refining soil structure, culminating in amplified plant growth and productivity [21]. There remains a crucial need to thoroughly examine the potential of deashed biochar as an amendment against drought stress and Cr toxicity, warranting significant attention in research and application.

Strigolactone is pivotal in enhancing crop resilience amid drought by promoting crucial root development, enabling plants to explore deeper soil layers for water and nutrients [22]. They regulate stomatal closure, which is vital for reducing water loss via transpiration while maintaining essential gas exchange for photosynthesis [23]. Moreover, strigolactone bolsters overall stress tolerance by synthesizing stress-related proteins and compounds, significantly improving plant endurance in adverse conditions [24].

Radish is a nutritious vegetable rich in vitamins (C and B-complex), minerals (potassium, calcium, iron), and dietary fiber, offering antioxidants like anthocyanins with potential health benefits [25]. It adds diversity, flavor, and color to meals, making it a popular addition to various dishes. Agriculturally, radishes are significant due to their short cultivation period and year-round growth, serving as a valuable rotational crop, enhancing soil health, and breaking disease cycles [26]. In crop rotation, radishes aid in weed and pest control, soil improvement, and nutrient enrichment for subsequent crops. They are also environmentally crucial as bio-accumulators, helping soil remediation [27]. Chromium can impact radish production by causing heavy metal uptake, direct toxicity, growth inhibition, nutrient imbalances, disruption of soil ecosystems, and food safety concerns, mainly if Cr(VI) contamination occurs in radish roots, posing health risks to consumers [28].

This study aims to investigate an innovative approach to mitigate the impact of Cr contamination on radish cultivation. We hypothesize that combining deashed biochar and Strigolactone can improve radish growth and reduce the accumulation of Cr in radish tissues. This research aims to assess the effectiveness of deashed biochar and Strigolactone in reducing Cr uptake by radishes, enhancing radish growth, and improving the quality of radish crops. By addressing the current knowledge gap and proposing a sustainable and eco-friendly solution to mitigate the adverse effects of Cr contamination on radish cultivation, this research contributes to the broader goal of safeguarding ecosystems, crops, and human health.

## Material and methods

### Experimental site and climate

In 2022, a pot study was done at the research area of ResearchSolution, located at 30°09'41.6"N 71°36'38.0"E. The climate of the area was semi-arid.

### Biochar preparation

Waste materials sourced from a local market, situated at coordinates 30°11'29.8''N and 71°28'48.8''E, were utilized to produce biochar. The collected waste was initially sun-dried and subsequently cut into small pieces. These prepared waste materials underwent pyrolysis under aerobic conditions at a controlled temperature of 325±5°C. The physicochemical properties of the biochar generated in the pre-experimental phase are summarized in Table 1. Following the pyrolysis process, the material was allowed to cool before undergoing crushing and grinding to obtain particles with a size of <2 mm. The resultant biochar was then stored in plastic containers for future use in biochar production.

**Table 1:** Pre-experimental soil, biochar, and irrigation characteristics

Soil	Values	Biochar	Values	Irrigation	Values
pH	8.05	pH	7.07	pH	6.02
EC*e* (dS/m)	2.97	EC*e* (dS/m)	1.43	EC*e* (dS/m)	216
SOM (%)	0.62	Ash Content (%)	2.42	Carbonates (meq./L)	0.00
TN (%)	0.031	Volatile Matter (%)	16.82	Bicarbonates (meq./L)	3.16
EP (mg/kg)	10.11	Fixed carbon (%)	80.76	Chloride (meq./L)	0.12
AK (mg/kg)	165	TN (%)	0.18	Ca+Mg (meq./L)	1.02
Silt (%)	42	TK (%)	0.045	Sodium (mg/L)	167
Sand (%)	29	TP (%)	0.098	RSC (meq./L)	2.14
Clay (%)	36	Surface area (m^2^/g)	355	TN (Total Nitrogen)	
Texture	Clay Loam	CEC (meq./ 100 g)	413	EP (Extractable Phosphorus)	
				AK (Available Potassium)	
				CEC (Cation Exchange Capacity)	
				EC (Electrical Conductivity)	

### Deashing biochar

To create the biochar, it was initially washed using tap water to eliminate any impurities. After removing the ash content, the biochar was subjected to meticulous rinsing with deionized water to completely remove any residual ash residues. The biochar was allowed to air-dry in a well-ventilated environment until it was dry. Subsequently, the deashed biochar was appropriately stored for future utilization [12, 29].

### Strigolactone

A solution of strigolactone with a concentration of 20µM was made by precisely measuring 0.012 g of the compound, considering its molar mass to be approximately 320 g/mol. This specific amount was dissolved thoroughly in 1000 mL of analytical-grade methanol, guaranteeing its full dissolution within the solvent.

### Seed collection, sterilization, and sowing

The radish seeds utilized in this research were acquired from an authorized seed dealer of the Government of Punjab, Pakistan. The chosen seeds underwent a surface sterilization process before sowing. This procedure entailed subjecting the seeds to a 5% sodium hypochlorite solution and three subsequent rinses with 95% ethanol. After the sterilization, the seeds underwent three washes with sterilized deionized water to ensure the removal of any residual sterilizing agents. Subsequently, ten seeds were planted in each pot containing 15 kg of soil. Following germination, a thinning process was employed to retain two seedlings in each pot [30].

### Soil sampling and analysis

For characterization, a composite soil sample (made from 4 replicates) was subjected to air-drying and passed through a 2-mm sieve [31]. Standard protocols were adopted for soil pH and EC [32, 33]. For organic matter determination, potassium dichromate and ferrous ammonium sulfate were used [34]. However, soil total N [35], available P [36], and extractable K [37] were also analyzed using standard protocols. Particle size analysis was done using a hydrometer and USDA textural triangle [38].

### Experimental design and treatment plan

The experiment followed a completely randomized design (CRD), incorporating four replications for every treatment. The treatment plan includes control without any treatment, 20µM Strigolactone, 0.75%DAB, and 20µM Strigolactone + 0.75%DAB. Furthermore, there were two different Cr stress levels applied, denoted as control (0Cr) and 200Cr (200 mg Cr/kg soil) [39]. For inducing artificial Cr toxicity, we utilized analytical-grade potassium dichromate salt (K_2_Cr_2_O_7_) following the method outlined by Danish et al. [40].

The K_2_Cr_2_O_7_ used in this study was acquired with Product Number 207802, sourced from Batch Number MKCT4740, and produced by the SIGALD brand. The chemical has a CAS Number of 7778-50-9 and an MDL Number of MFCD00011367. Its molecular formula is Cr2K2O7, with a formula weight of 294.18 g/mol. The 200 mg Cr/kg soil toxic level was selected due to the toxicity range (150 mg Cr/kg soil) observed by [41].

### Pot preparation and sowing

A plastic container with dimensions of 15 inches in width and 45 inches in depth was loaded with 10 kg of soil. The initial physicochemical characteristics of the soil before the experiment are outlined in Table 1. In each container, ten seeds were sown, and, following a period of seven days from germination, two robust seedlings were retained after thinning.

### Fertilizer and irrigation

At the time of sowing, it's recommended to combine well-decomposed cow dung with specific quantities of nutrients per acre in the soil: nitrogen at a rate of 25kg (using urea at 55kg/acre = 0.68g/pot) and phosphorus at 12kg (utilizing SSP at 75kg/acre = 0.15g/pot). In each pot 50% moisture was maintained based on field capacity (w/w).

### Harvesting and data collection

The shoot and root lengths were assessed through manual scale measurements, and the plant's fresh and dry weights were measured through a weighing balance.

### Relative water content

To assess the relative water content (RWC) of freshly harvested leaves, we employed the procedure outlined by [42]. Initially, leaf samples were collected, and their initial fresh weight (FW) was recorded. These leaf samples were then immersed in Petri dishes filled with distilled water, allowing them to absorb water until reaching full turgidity, and the resulting turgid weight (TW) was documented. Following the turgidity phase, the leaf samples were subjected to oven drying, and their final dry weight (DW) was determined. The RWC was calculated using the following formula:

$$\mathrm{RWC }(\mathrm{\%}) = (\mathrm{FW }-\mathrm{ DW}) / (\mathrm{TW }-\mathrm{ DW}) \times 100$$

### Chlorophyll contents and carotenoids

We followed a procedure of Arnon method to assess chlorophyll a, chlorophyll b, and total chlorophyll levels in freshly harvested wheat leaves [43]. The extraction process involved using an 80% acetone solution, and absorbance measurements were conducted at distinct wavelengths: 663 nm for chlorophyll a, 645 nm for chlorophyll b, and 470 nm for carotenoids.

$$\begin{array}{c}\mathrm{Chlorophyll a }\left(\frac{{\text{mg}}}{{\text{g}}}\right)=\frac{\left(12.7 \times \mathrm{ A}663\right)-\left(2.69 \times \mathrm{ A}645\right)\times {\text{V}}}{1000 \times {\text{W}}}\\ \mathrm{Chlorophyll b }\left(\frac{{\text{mg}}}{{\text{g}}}\right)=\frac{\left(22.9 \times \mathrm{ A}645\right)-\left(4.68 \times \mathrm{ A}663\right)\times {\text{V}}}{1000 \times {\text{W}}}\\ \begin{array}{c}\mathrm{Total Chlorophyll }\left(\frac{{\text{mg}}}{{\text{g}}}\right)=\frac{20.2\left({\text{A}}645\right)+8.02\left({\text{A}}663\right)\times {\text{V}}}{1000 \times {\text{W}}}\\ \mathrm{Carotenoids }\left(\frac{{\text{mg}}}{{\text{g}}}\right)={\text{OD}}480+0.114 (\mathrm{OD }663)-0.638 (\mathrm{OD }645)\end{array}\end{array}$$

### Antioxidants

We evaluated SOD activity by measuring the inhibition of nitro blue tetrazolium (NBT) at 560 nm [44]. POD activity was determined at 420 nm by following the protocol of [45]. CAT activity was assessed by measuring the decline in absorbance at 240 nm due to H_2_O_2_ decomposition [46]. In the case of APX activity, oxidation of ascorbate was assessed in the presence of H_2_O_2_ at 290 nm [47]. To determine the level of MDA, we checked the sample extract by reacting it with thiobarbituric acid (TBA), forming a colored complex. The absorbance of this complex was measured at 532nm, and the MDA content was subsequently calculated [48]. The measurement of glutathione reductase (GR) activity was done at 340 nm [49].

### Determination of nonenzymatic antioxidants

To assess glutathione (GSH) content, an equivalent volume of 5% sulfosalicylic acid (w/v), 100 mM phosphate buffer (pH 7.0) and 5.5-dithiobis (2-nitrobenzoic acid) were used. The final absorbance was measured spectrophotometrically at 412 nm [50]. For the determination of ascorbate (AsA) 10% trichloroacetic acid was used. The final absorbance was taken at 525 nm [51].

### Electrolyte leakage

One gram uniform size leaves were collected using a 1 cm diameter steel cylinder. These leaf sections were added in test tubes having 20 ml of deionized water and incubated at 25 °C for 24 hours. Following this incubation, the 1^st^ electrical conductivity (EC1) was noted using a pre-calibrated EC meter. After that test tubes were heated in a water bath at 120 °C for 20 minutes, and 2^nd^ electrical conductivity (EC2) was recorded. Electrolyte leakage was calculated using following equation [52].

$$\mathrm{Electrolyte Leakage }\left(\mathrm{\%}\right)=\left(\frac{{\text{EC}}1}{{\text{EC}}2}\right)\times 100$$

### Free proline

Free proline was quantified using sulfosalicylic acid, glacial acetic acid and ninhydrin solutions [53]. The mixture was heated at 100 °C and added 5 ml of toluene. The absorbance was measured at 520 nm.

### Statistical analysis

The data were subjected to standard statistical analysis [54], including a two-way ANOVA, to evaluate the significance of the treatments. To compare the treatments, paired comparisons were conducted using the Tukey test with a significance level set at p ≤ 0.05. Furthermore, cluster plots, convex hull plots, and pearson correlation analysis was assessed by using OriginPro software [55].

## Results

### Shoot and root length, plant fresh and dry weight

**Fig. 1:**
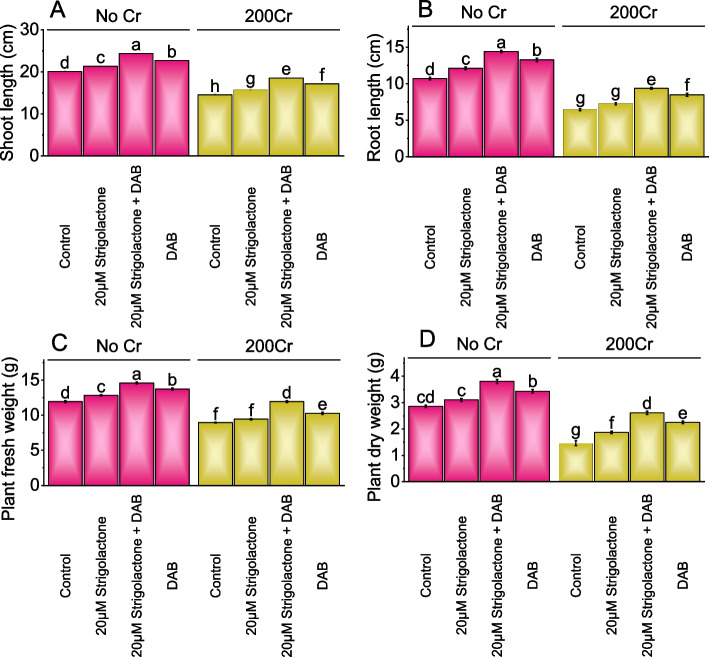
Effect of treatments on shoot length (**A**), root length (**B**), plant fresh weight (**C**), and plant dry weight (**D**) of radish cultivated under no Cr stress and 200Cr stress. The bars represent the means of four replicates with standard error. The Tukey test revealed significant changes at p < 0.05, shown by the different letters on the bars

The control group had a mean shoot length of 20.11 cm with no Cr stress. The application of 20µM Strigolactone resulted in a modest 5.97% increase in shoot length over the control, and the combination of 20µM Strigolactone and DAB showed a more substantial 21.03% increase without Cr stress. Compared to the control in No Cr stress, the addition of DAB alone exhibited a 12.85% increase in shoot length. In 200Cr stress, the treatment of 20µM Strigolactone showed a 7.91% increase in shoot length, and 20µM Strigolactone+DAB combination led to the highest increase of 27.29%, while the treatment with DAB alone resulted in an 18.19% increase over the control (Fig. 1A; S1).

In No Cr, the application of 20µM Strigolactone led to a 13.06% increase in root length, and 20µM Strigolactone+DAB treatment resulted in a significant 34.55% rise contrasted to the control. Similarly, DAB treatment alone showed a 23.84% increase compared to the control. Under 200Cr, in contrast to the control, applying 20µM Strigolactone increased root length by 12.72%, while the combination of 20µM Strigolactone and DAB exhibited a remarkable 45.60% increase. DAB treatment showed a significant 31.72% increase related to the 200Cr stressed control group (Fig. 1B; S1).

The plant's fresh weight showed a 7.46% increase with the treatment of 20µM Strigolactone, which further increased to 22.22% when 20µM Strigolactone and DAB treatment were applied. When only DAB treatment was applied, there was a 15.02% increase in plant fresh weight under no Cr stress. Under 200Cr stress, the addition of 20µM Strigolactone treatment resulted in a 5.31% increase related to the control, and this increase further rose to 33.25% when 20µM Strigolactone and DAB were introduced. When DAB treatment was applied, a 14.63% increase in fresh weight was evaluated to the 200Cr stressed control (Fig. 1C).

In No Cr, adding 20µM Strigolactone resulted in an 8.59% increase in the plant's dry weight compared to the control. When 20µM Strigolactone was applied along with DAB, the plant dry weight exhibited a substantial 33.18% increase contrasted to the control, and the application of DAB treatment in the absence of chromium led to a 20.16% increase in plant dry weight. In the presence of 200Cr, adding 20µM Strigolactone treatment resulted in a 28.19% increase in plant dry weight related to the control. When 20µM Strigolactone was combined with DAB in the 200Cr stress, the plant dry weight showed a remarkable 78.91% increase, and the application of DAB treatment in the presence of 200Cr led to a substantial 54.10% increase in plant dry weight (Fig. 1D).

### Chlorophyll and carotenoid content

**Fig. 2:**
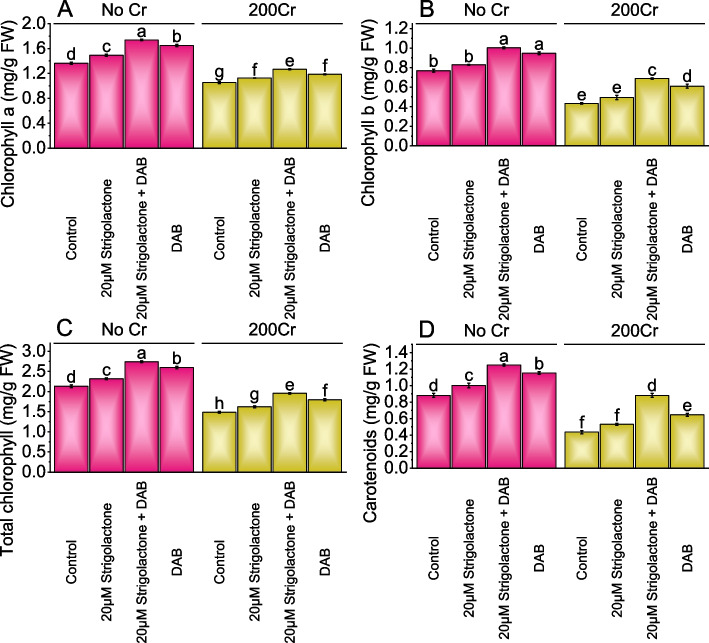
Effect of treatments on chlorophyll a (**A**), chlorophyll b (**B**), total chlorophyll (**C**), and carotenoid (**D**) of radish cultivated under no Cr stress and 200Cr stress. The bars represent the means of four replicates with standard error. The Tukey test revealed significant changes at p < 0.05, shown by the different letters on the bars

Chlorophyll a content in the control group had an average value of 1.36 mg/g under No Cr. The addition of 20µM Strigolactone resulted in a 9.22% increase in chlorophyll a content as opposed to the control, and the combination of Strigolactone and DAB showed a more significant 27.41% increase. The DAB treatment under No Cr stress conditions led to a 20.82% increase in chlorophyll a content. In 200Cr stress condition, the 20µM Strigolactone treatment showed a 6.99% increase in chlorophyll a content over the control. The combination of Strigolactone and DAB further improved the chlorophyll a content with a 20.41% rise. The DAB treatment under 200Cr stress conditions resulted in a 12.66% improvement in chlorophyll a content compared to the control (Fig. 2A).

In No Cr, chlorophyll b content increased by 7.73% with 20µM Strigolactone, 30.26% when combined with 20µM Strigolactone+DAB, and 23.07% with DAB treatment. Under chromium stress conditions (200Cr), chlorophyll b content increased by 14.00% with 20µM Strigolactone treatment, 58.53% when combined with 20µM Strigolactone+DAB treatment, and 40.67% with DAB treatment (Fig. 2B).

The total chlorophyll content increased by 8.68% when treated with 20µM Strigolactone over the control. When 20µM Strigolactone+DAB treatment was applied, there was a substantial 28.44% increase in chlorophyll content related to the control, and DAB treatment also resulted in a notable 21.63% rise in chlorophyll content. For the 200Cr stress, a 9.03% increase in chlorophyll content was observed with the addition of 20µM Strigolactone related to the control, and the combination of 20µM Strigolactone and DAB led to a significant 31.54% increase in total chlorophyll content. When DAB treatment was applied, there was 20.84% increase in chlorophyll content related to the 200Cr stressed control (Fig. 2C).

In No Cr, 20µM Strigolactone treatment raised carotenoid content by 13.76%, and when 20µM Strigolactone combined with DAB, it resulted in a substantial 41.70% increase over the control. Treatment DAB led to a 30.75% increase in carotenoid content contrasted to the control. In the presence of 200Cr, 20µM Strigolactone increased carotenoid content by 21.63%, and the 20µM Strigolactone combination with DAB resulted in a significant 102.24% increase. Application of DAB treatment showed a considerable 48.19% increase in carotenoid content contrasted to the 200Cr stressed control (Fig. 2D).

### Relative water content (RWC), protein content, and electrolyte leakage

**Fig. 3:**
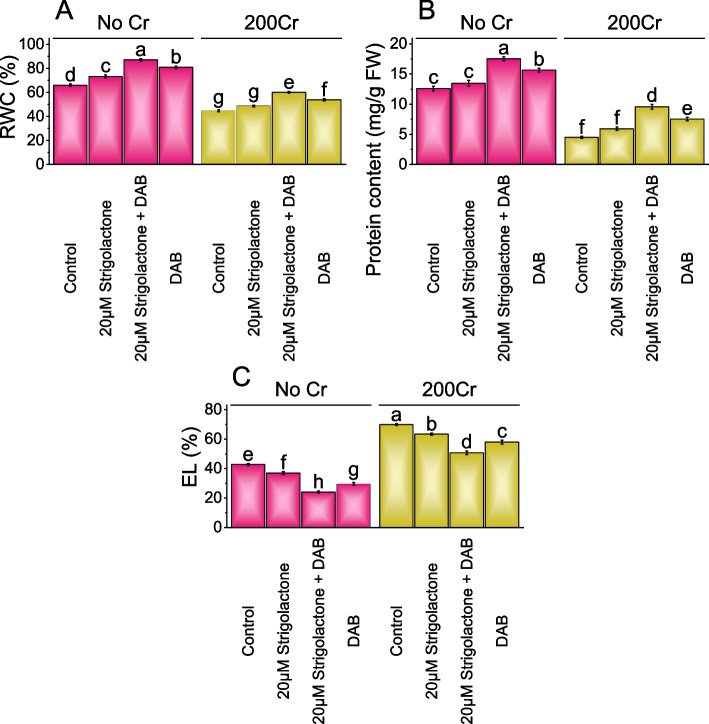
Effect of treatments on Relative water content (RWC) (**A**), protein content (**B**), and Electrolyte leakage (EL) (**C**) of radish cultivated under no Cr stress and 200Cr stress. The bars represent the means of four replicates with standard error. The Tukey test revealed significant changes at p < 0.05, shown by the different letters on the bars

The addition of 20µM Strigolactone led to an 11.06% increase in RWC from the control. In comparison, the inclusion of DAB along with Strigolactone resulted in a substantial 31.90% boost in RWC in No Cr. The DAB treatment showed a 22.54% increase in RWC from the control. Under 200Cr stressed conditions, adding 20µM Strigolactone resulted in an 8.81% increase in RWC related to the control, and combined with DAB, Strigolactone treatment resulted in a significant 34.38% increase in RWC. The DAB treatment led to a significant 20.13% increase in RWC than the 200Cr stressed control (Fig. 3A).

The protein content in the No Cr stress showed a 6.83% increase when treated with 20µM Strigolactone and a substantial 39.18% increase when 20µM Strigolactone treatment was combined with DAB. The addition of DAB treatment resulted in a 24.20% increase in protein content related to the control. In 200Cr stress, there was a 31.94% increase in protein content when 20µM Strigolactone treatment was applied and a remarkable 112.72% increase with 20µM Strigolactone+DAB treatment. The DAB treatment exhibited a 67.61% rise in protein content related to the 200Cr stressed control (Fig. 3B).

In No Cr, the application of 20µM Strigolactone led to a 16.30% decrease in EL (Electrolyte leakage), and the combined treatment of 20µM Strigolactone and DAB resulted in a significant 78.07% reduction in EL related to the control. When DAB treatment was applied in the absence of chromium, there was a 44.64% decrease in EL. In the presence of 200Cr stress, 20µM Strigolactone led to a 10.32% decrease in EL, and the combined treatment of 20µM Strigolactone and DAB caused a 37.83% reduction in EL compared to the 200Cr stressed control. In the presence of 200Cr, adding DAB treatment resulted in a 20.75% decrease in EL evaluated to the 200Cr stressed control (Fig. 3C).

### Proline content, H_2_O_2_ and MDA

**Fig. 4:**
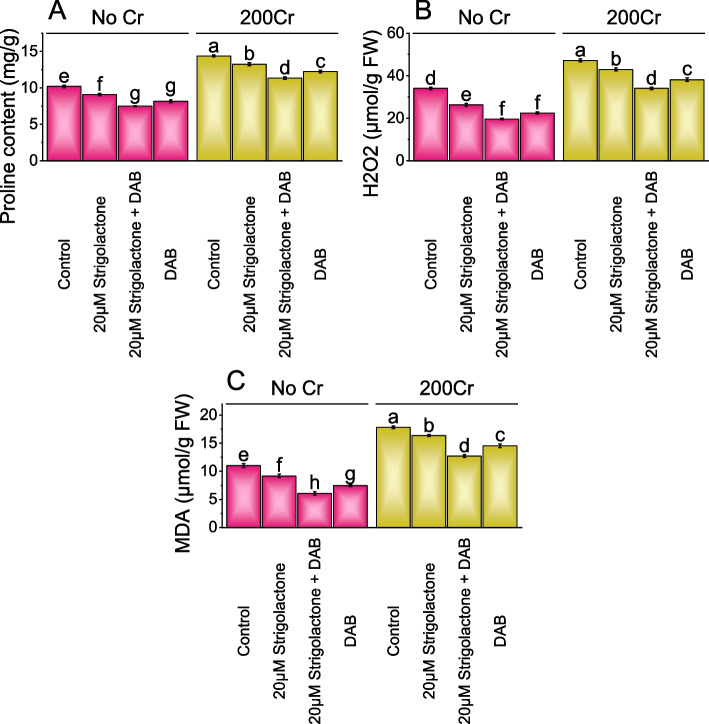
Effect of treatments on proline content (**A**), H_2_O_2_ (**B**), and MDA (Malondialdehyde) (**C**) of radish cultivated under no Cr stress and 200Cr stress. The bars represent the means of four replicates with standard error. The Tukey test revealed significant changes at p < 0.05, shown by the different letters on the bars

In No Cr, 20µM Strigolactone treatment resulted in a 12.27% decrease in proline content more than the control, and the addition of DAB along with Strigolactone led to a more substantial 36.16% decrease. When only DAB treatment was applied, proline content decreased by 24.99% compared to the control. In 200Cr stress, 20µM Strigolactone treatment showed an 8.49% decrease in proline content and combining Strigolactone with DAB resulted in a 26.80% reduction. The addition of DAB treatment in the 200Cr stress led to a 17.44% decrease in proline content (Fig. 4A).

When 20µM Strigolactone was added in No Cr, H_2_O_2_ levels decreased by 29.54%, and when Strigolactone and DAB were combined, H_2_O_2_ levels decreased by a significant 73.06% in comparison to the control. The treatment DAB alone showed a 51.20% decrease in H_2_O_2_ levels more than the control. In 200Cr stress, the addition of 20µM Strigolactone resulted in a more modest 9.90% decrease, and the combination of 20µM Strigolactone and DAB showed a 38.70% decrease as opposed to the control to the control. Compared to the control, in the 200Cr stressed condition, H_2_O_2_ levels decreased by 23.76% with the addition of DAB treatment (Fig. 4B).

In No Cr, treatment of 20µM Strigolactone reduced MDA levels by 20.28% from the control and combining 20µM Strigolactone with DAB resulted in an 81.88% decrease. The DAB treatment reduced MDA levels by 48.13% without stress (No Cr). In chromium (200Cr) stress, 20µM Strigolactone decreased MDA levels by 8.93% in contrast to the control, and when combined with 20µM Strigolactone+DAB, there was a 40.51% decrease. Adding DAB treatment in the presence of chromium led to a 22.72% decrease in MDA levels above the control (Fig. 4C).

### SOD, POD, CAT, and APX activity

**Fig. 5:**
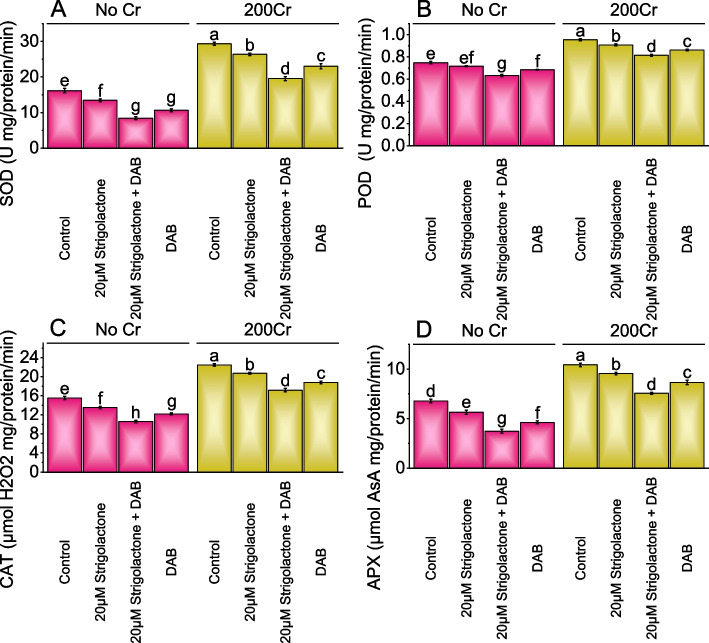
Effect of treatments on SOD (Superoxide dismutase) (**A**), POD (Peroxidase) (**B**), CAT (Catalase) (**C**), and APX (Ascorbate peroxidase) (**D**) of radish cultivated under no Cr stress and 200Cr stress. The bars represent the means of four replicates with standard error. The Tukey test revealed significant changes at p < 0.05, shown by the different letters on the bars

When compared to the control group, the addition of 20µM Strigolactone treatment No Cr led to a 20.37% decrease in SOD activity; when DAB was added with 20µM Strigolactone, there was a substantial 92.26% reduction in SOD activity. The addition of DAB treatment in the No Cr group resulted in a 52.29% decrease in SOD activity. In the 200Cr stress, the introduction of 20µM Strigolactone treatment showed an 11.28% decrease in SOD activity over the control. When 20µM Strigolactone was combined with DAB in the 200Cr stressed group, SOD activity decreased by 50.04%, and the addition of DAB treatment led to a 27.36% decrease in SOD activity contrasted to the 200Cr control (Fig. 5A).

In No Cr stress over the control, the addition of 20µM Strigolactone treatment showed a 4.36% decrease in peroxidase (POD), while the addition of 20µM Strigolactone+DAB treatment intensified the reduction to 18.26%. Meanwhile, the No Cr treatment with DAB alone resulted in a 9.16% decrease in POD activity. In the 200Cr stressed group, treatment of 20µM Strigolactone led to a 5.12% decrease in POD activity, and the addition of 20µM Strigolactone+DAB resulted in a more significant 17.12% decrease parallel to the baseline. Treatment DAB exhibited a 10.67% decrease in POD activity, demonstrating the impact of different treatments on POD activity (Fig. 5B).

Applying 20µM Strigolactone alone in No Cr demonstrated a 14.92% drop-in CAT activity compared to the control. When DAB was added along with 20µM Strigolactone, CAT activity dropped significantly, showing a 46.83% decrease contrasted to the control, and the DAB treatment led to a 27.37% reduction in CAT activity. Under 200Cr stress, the control exhibited a 22.46 µmol H2O2 mg/protein/min CAT activity level. The addition of 20µM Strigolactone under 200Cr stress resulted in an 8.58% decrease in CAT activity compared to the control. In the 200Cr stressed condition, the application of DAB alone resulted in a 19.50% drop-in CAT activity, and the combination of DAB and 20µM Strigolactone caused a 30.89% decline in CAT activity relative to the control (Fig. 5C).

In No Cr, the control group exhibited an APX activity of 6.78 µmol AsA mg/protein/min, and the addition of 20µM Strigolactone treatment resulted in a 20.02% decrease. The combination of 20µM Strigolactone with DAB led to a significant decrease of 81.76% in APX activity above the control, and DAB treatment produced a 46.96% reduction in APX activity. When chromium stress was present (200Cr), the control group showed an APX activity of 10.44 µmol AsA mg/protein/min. The addition of 20µM Strigolactone resulted in a 9.35% decrease in APX activity compared to the control, while the combination of 20µM Strigolactone with DAB showed a 38.25% decrease. Additionally, DAB treatment in the presence of chromium led to a 20.56% decrease in APX activity linked to the 200Cr stressed control (Fig. 5D).

### GR, GSH, and ASA activity

**Fig. 6:**
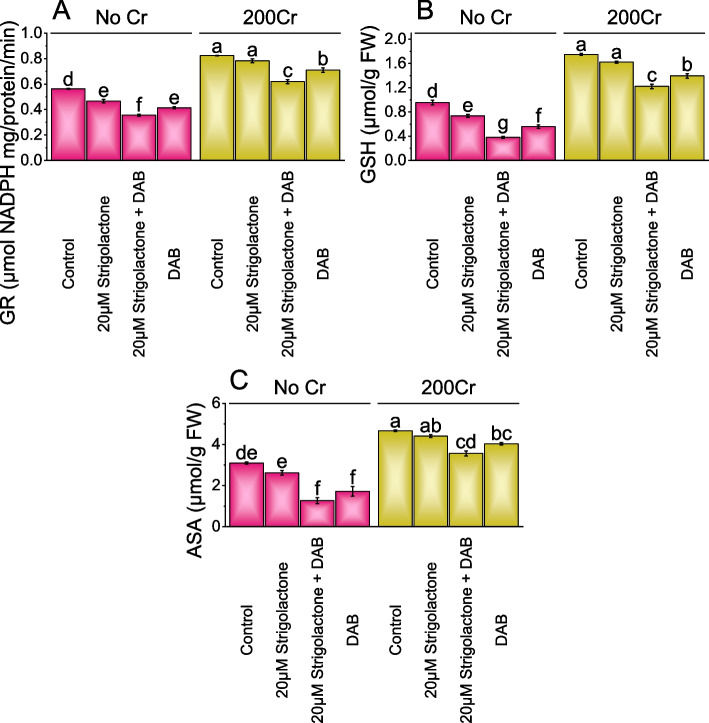
Effect of treatments on GR (Glutathione Reductase) (**A**), GSH (Glutathione) (**B**), and APX (Ascorbic acid) (**C**) of radish cultivated under no Cr stress and 200Cr stress. The bars represent the means of four replicates with standard error. The Tukey test revealed significant changes at *p* < 0.05, shown by the different letters on the bars

Under the no Cr stress, the addition of 20µM Strigolactone treatment resulted in a 20.77% decrease in GR activity, with a more significant 57.89% decrease when 20µM Strigolactone combined with DAB, and DAB treatment led to a 35.79% reduction in GR activity in comparison to the control group. Under 200Cr stress, GR activity decreased by 5.21% when 20µM Strigolactone treatment was added, a more substantial 32.98% reduction when 20µM Strigolactone combined with DAB, and a 16.11% decrease when DAB was applied alone in contrast to the control treatment (Fig. 6A).

In No Cr stress, the addition of 20µM Strigolactone resulted in a 29.90% decrease in GSH levels related to the control, while the combination of 20µM Strigolactone and DAB led to a substantial 150.85% decrease in GSH levels. In contrast, the treatment with DAB alone showed a 71.36% decrease in GSH levels without any stress applied. In chromium (200Cr) stress, adding 20µM Strigolactone resulted in a significant 7.82% decrease in GSH levels related to the control. The combination of 20µM Strigolactone and DAB showed a 43.03% decrease in GSH levels, and the treatment with DAB alone exhibited a 25.27% decrease in GSH levels compared to the 200Cr stressed control (Fig. 6B).

Under No Cr, adding 20µM Strigolactone resulted in a notable 18.31% decrease in ASA levels compared to the control. In comparison to the control without any stress, the combination of 20µM Strigolactone and DAB, ASA levels showed a significant 144.79% decrease, and DAB treatment alone caused an 80.58% decrease in ASA. Under 200Cr stress conditions, the 20µM Strigolactone treatment led to a modest 5.99% decrease in ASA when compared to the control, while the combination of 20µM Strigolactone and DAB showed a 31.04% reduction. In comparison to the 200Cr stressed control, the DAB treatment showed a 15.77% drop in ASA levels (Fig. 6C).

### Cr uptake in radish and leaves

In comparison to the control under no Cr, the application of 20µM strigolactone decreased Cr uptake in radish by 16.89%, while 20µM strigolactone+DAB resulted in an 84.21% reduction, and DAB alone led to a 42.49% decrease. Under 200 Cr stress, adding 20µM strigolactone decreased Cr uptake in radish by 23.53%, and 20µM strigolactone+DAB resulted in a significant 98.35% reduction. Treatment DAB alone led to a 74.45% decrease in Cr uptake in radish over the Cr-stressed control (Table 2).

**Table 2:** Effect of treatments on Cr uptake in radish and leaf

**Treatment**	**Cr in Radish (µg/g)**	**Cr in leaf (µg/g)**
	**No Cr**	
Control	6.45e	4.72e
20µM Strigolactone	5.36ef	4.11ef
20µM Strigolactone + DAB	3.50f	2.40g
DAB	4.53ef	3.21fg
	**200 Cr**	
Control	30.43a	8.78a
20µM Strigolactone	23.31b	7.64b
20µM Strigolactone + DAB	13.57d	5.66d
DAB	17.45c	6.68c

Values are the mean of 4 replicates. Different letters showed significant changes at *p*≤ 0.05; Tukey Test. No Cr; 200 Cr

In No Cr stress, introducing 20µM strigolactone showed a significant 14.83% decrease in leaf Cr uptake, and 20µM strigolactone+DAB exhibited a 96.77% reduction in contrast to the control. In comparison to the control under no Cr, adding DAB treatment resulted in a 47.00% drop in leaf Cr uptake. When 200 Cr was introduced, the 20µM strigolactone showed a 14.88% decrease, and the combined application of 20µM strigolactone+DAB signified a 55.06% reduction in leaf Cr uptake over the control. The introduction of DAB alongside 200 µM Cr led to a 31.44% decrease evaluated to the 200 Cr-stressed control (Table 2).

### Convex hull and hierarchical cluster analysis

**Fig. 7:**
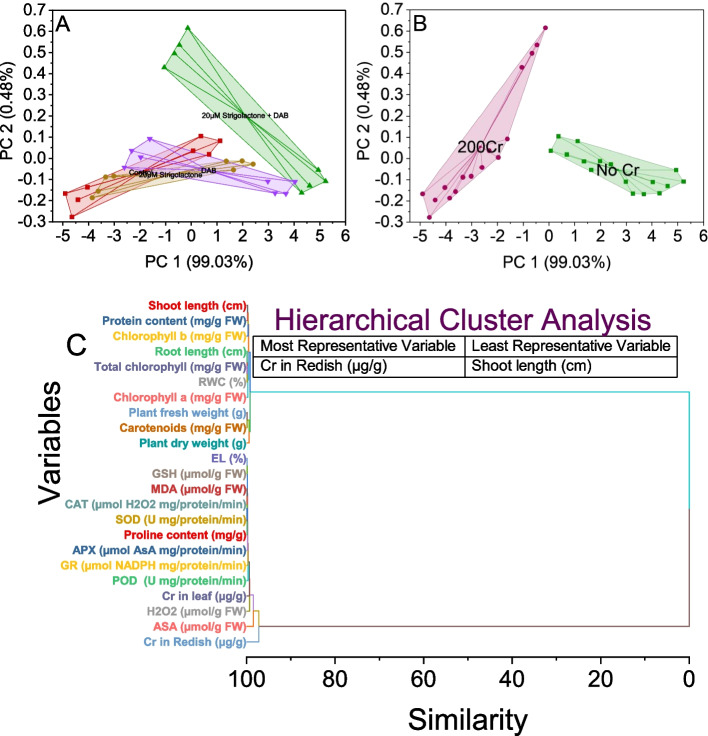
Cluster plot convex hull for growth attributes (Shoot and root length, Plant fresh and dry weight, Chlorophyll content, Carotenoids content, RWC, and total protein) treatments (**A**), Cr levels (**B**), and hierarchical cluster plot (**C**) for studied attributes

The convex hull analysis was performed on a dataset with two principal components, PC1 and PC2, representing different treatment conditions. In the control group, the Convex Hull encompassed a large area, from 0.06921 to 4.29289 on PC1 and from -0.27729 to 0.42932 on PC2. When treated with 20µM Strigolactone, the Convex Hull narrowed, indicating a reduction in the data spread, as it ranged from 1.35986 to 2.40387 on PC1 and from -0.05497 to -0.01327 on PC2. This trend continued when 20µM Strigolactone was combined with DAB, further tightening the Convex Hull, showing a range from 4.29289 to 5.22778 on PC1 and from -0.16328 to -0.0553 on PC2. In contrast, the DAB treatment alone exhibited a Convex Hull that was separated from the other treatments, covering a PC1 range from 2.97952 to 4.03403 and a PC2 range from -0.16727 to -0.04296 (Fig. 7A).

The convex hull analysis was performed on the given data, which represents stress-related scores in two principal components (PC 1 and PC 2) for two different conditions: No Cr and 200Cr. The analysis shows that in the No Cr condition, the data points form a convex hull that covers the range from approximately 0.01855 to 4.94379 in PC 1 and from -0.16492 to 0.10496 in PC 2. The stress-related scores within this condition exhibit a compact distribution within this convex hull. In contrast, under the 200Cr condition, the data points from another convex hull that spans a broader range, from approximately -4.90501 to -0.13653 in PC 1 and from -0.27729 to 0.61557 in PC 2. This indicates that the 200Cr condition results in a more scattered distribution of stress-related scores, suggesting greater variability in response to stress compared to the No Cr condition (Fig. 7B).

The hierarchical cluster analysis was conducted on a dataset consisting of various variables, measuring different physiological parameters. The analysis revealed distinct groupings based on the similarity of these variables. One prominent cluster includes variables 13 and 14, which represent MDA and CAT, showing a high degree of similarity (0.08608). These variables share a common characteristic in terms of their response. Another cluster combines variables 15, 22, and 23, representing SOD, an unspecified variable, and an unspecified variable, respectively. Variable 15, representing SOD, exhibits a similarity of 0.09682 with variable 23, suggesting a potential relationship in their responses.

Additionally, variables 5 and 6, representing Total chlorophyll and RWC, respectively, have a similarity of 0.10138, indicating their potential co-occurrence in the response pattern. Another cluster encompasses variables 11 and 12 representing EL and GSH, respectively. These two variables show a similarity of 0.11225, suggesting a shared response to certain conditions. Further analysis reveals clusters involving variables related to proline content, root length, and plant parameters like fresh weight, carotenoids, shoot length, and protein content. In contrast, variables 21 (ASA) and 40 (H_2_O_2_) exhibit the highest similarity (1.46039), indicating a strong association between ASA and H_2_O_2_ in the physiological response. Lastly, there are two groups of variables, 39 and 40, which have an extremely high similarity (99.24501 and 98.53961, respectively), implying that they are nearly identical in their responses, possibly representing the same measurement under different conditions (Fig. 7C).

### Pearson correlation analysis

The Pearson correlation analysis was conducted to assess the relationships between various physiological parameters. The results indicate strong positive correlations between several variables. For instance, shoot length (cm) showed a high positive correlation with root length (cm) (r = 0.99683), plant fresh weight (g) (r = 0.98835), and plant dry weight (g) (r = 0.99348). These results suggest that these variables are tightly associated, with increases in one variable generally corresponding to increases in the others. Chlorophyll a, chlorophyll b, and total chlorophyll also displayed strong positive correlations with one another, with coefficients of 0.98696, 0.99464, and 0.99501, respectively. Carotenoids exhibited positive correlations with most of the chlorophyll-related variables, emphasizing their interrelatedness. Relative Water Content (RWC) had high positive correlations with shoot length, root length, and several chlorophyll-related variables, indicating a strong connection between RWC and these parameters.

**Fig. 8:**
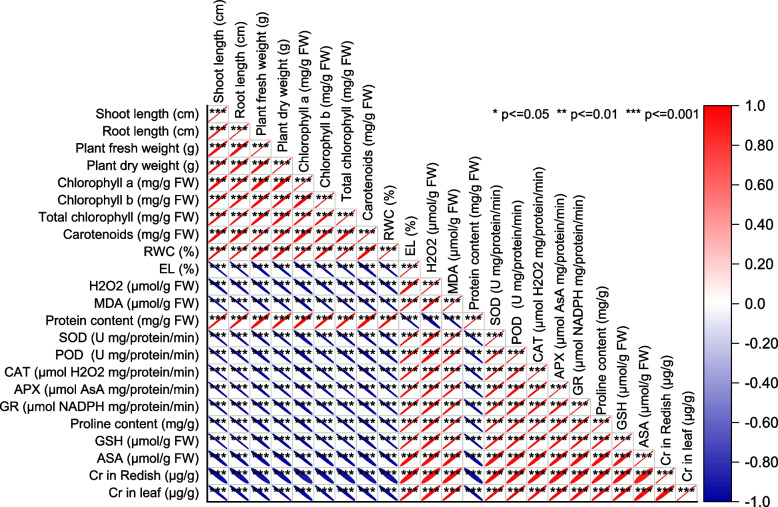
Pearson correlation for studied attributes

Conversely, electrolyte leakage (EL) demonstrated strong negative correlations with the variables, with coefficients around -0.9979, suggesting an inverse relationship. Hydrogen peroxide (H_2_O_2_) and malondialdehyde (MDA) exhibited negative correlations with various parameters, reflecting potential relationships in response to stress. Protein content displayed positive correlations with several variables, highlighting its potential association with these physiological parameters. Enzyme activities such as superoxide dismutase (SOD), peroxidase (POD), catalase (CAT), ascorbate peroxidase (APX), and glutathione reductase (GR) also demonstrated a mix of positive and negative correlations with other variables, indicating their involvement in the overall physiological response to stress. Proline content and glutathione (GSH) showed negative correlations with most variables, suggesting potential roles in stress responses. Notably, ascorbic acid (ASA) displayed weaker correlations with other variables but still had some associations, particularly with shoot and root length, suggesting its involvement in specific aspects of the response (Fig. 8).

## Discussion

In this study, we investigated the impact of Strigolactone and deashed biochar (DAB) on radish growth under both non-stressed and chromium-contaminated soil conditions. Strigolactone is known for its crucial role in plant development and stress responses. Our research aimed to understand how the application of Strigolactone and DAB, individually or in combination, influenced various aspects of radish growth and stress tolerance. Strigolactone is a plant hormone crucial in plant development and stress responses [56]. When 20µM Strigolactone was applied, it increased growth in shoots and roots by promoting cell elongation and division. Strigolactone interacts with auxins, hormones responsible for cell elongation and differentiation, resulting in enhanced shoot and root growth [57]. When plants were under chromium stress (200Cr), Strigolactone treatment helped reduce the negative effects of stress, possibly by improving nutrient uptake and reducing oxidative stress. Additionally, treating plants with biochar resulted in significant growth enhancement by improving soil structure and water retention, providing essential nutrients, and reducing the bioavailability of toxic metals like chromium [12, 58–60]. Combining both Strigolactone and DAB treatments showed the most significant growth improvements, suggesting that their synergistic effects enhance nutrient availability, water retention, and stress mitigation, leading to remarkable increases in plant growth under both stressed and non-stressed conditions. Strigolactone application can enhance photosynthesis by promoting the development of chloroplasts, leading to increased chlorophyll content [61]. This hormone likely stimulates gene expression in chlorophyll biosynthesis and maintenance. Biochar has a high surface area and porous structure that can serve as a substrate for beneficial microorganisms [62–65]. These microorganisms can contribute to improved nutrient availability, root health, and overall plant performance, including chlorophyll synthesis [66]. Furthermore, biochar may adsorb and immobilize chromium, mitigating its toxic effects and allowing for increased chlorophyll production [67]. Strigolactone enhances plant stress tolerance by activating signaling pathways that improve antioxidant defenses, reducing oxidative damage caused by stressors like chromium [68]. This leads to better chlorophyll retention and photosynthetic activity. Biochar materials like DAB adsorb heavy metals, reducing their bioavailability and enhancing chlorophyll content, thus promoting plant health [69]. Strigolactone has been reported to stimulate the synthesis of secondary metabolites, including carotenoids, which serve as antioxidants and play vital roles in photoprotection [70]. By activating pathways related to carotenoid production, Strigolactone contributes to the increased carotenoid content observed in the study, as shown in (Fig. 2D). DAB's role in enhancing soil microbial activity can indirectly contribute to carotenoid enhancement. Beneficial microorganisms can improve nutrient uptake, increasing carotenoid production as these compounds are involved in photoprotection and antioxidant defense. Applying 20µM Strigolactone in combination with DAB enhances plant water status by improving root hydraulic conductivity and controlling stomatal aperture, reducing water loss, and enhancing water uptake. This treatment also increases protein content, potentially promoting synthesis and reducing degradation. It improves membrane stability, reduces electrolyte leakage, and decreases proline content, suggesting reduced stress and oxidative damage. Strigolactone and DAB together reduce H_2_O_2_ levels, indicating less oxidative stress and lower MDA levels, signifying reduced lipid peroxidation and membrane damage, collectively enhancing plant resilience [71]. The significant decrease in SOD activity when Strigolactone is combined with DAB is due to Strigolactone's influence on reactive oxygen species (ROS) regulation and DAB's inhibition of H2O2 breakdown. This disrupts the plant's ability to scavenge ROS effectively. Similarly, the reduction in POD activity is linked to Strigolactone's impact on plant signaling pathways, which DAB further intensifies [72]. CAT and APX activities are also negatively affected, with DAB intensifying oxidative stress. This disruption in the antioxidant defense system leads to increased oxidative damage in plant cells. Reductions in GR, GSH, and ASA levels are a result of Strigolactone and DAB affecting the plant's redox balance, compromising its ability to detoxify ROS and scavenge free radicals. Overall, the combination of Strigolactone and DAB disrupts the plant's antioxidant defense mechanisms and exacerbates oxidative stress.

## Conclusion

In conclusion, 0.75%DAB with 20µM strigolactone amendment effectively mitigates Cr stress. Applying 20µM Strigolactone with 0.75%DAB significantly improved radish growth and chlorophyll content under Cr stress. We encourage farmers to apply 20µM Strigolactone with 0.75%DAB to enhance radish resilience against Cr-contamination. Future research recommends that 20µM Strigolactone with 0.75%DAB be the best possible solution to mitigate Cr stress in radish under different agroclimatic conditions.

### Supplementary Information

**Supplementary file 1: Figure S1.** Effect of treatments on the growth of Radish under normal condition and Cr toxicity (200 mg Cr/kg soil).

## Data Availability

All data generated or analysed during this study are included in this published article.

## References

[1] Li F, Yang H, Ayyamperumal R, Liu Y (2022). Pollution, sources, and human health risk assessment of heavy metals in urban areas around industrialization and urbanization-Northwest China. Chemosphere.

[2] Syed A, Elgorban AM, Bahkali AH, Eswaramoorthy R, Iqbal RK, Danish S (2023). Metal-tolerant and siderophore producing Pseudomonas fluorescence and Trichoderma spp. improved the growth, biochemical features and yield attributes of chickpea by lowering Cd uptake. Sci Rep.

[3] Younis U, Danish S, Malik SA, Ahmed N, Munir TM, Rasheed MK. Role of cotton sticks biochar in immobilization of nickel under induced toxicity condition and growth indices of Trigonella corniculata L. Environ Sci Pollut Res. 2020;27. 10.1007/s11356-019-06466-3 31758478

[4] Azhar U, Ahmad H, Shafqat H, Babar M, Shahzad Munir HM, Sagir M (2022). Remediation techniques for elimination of heavy metal pollutants from soil: a review. Environ Res.

[5] Sana S, Ramzan M, Ejaz S, Danish S, Salmen SH, Ansari MJ (2024). Differential responses of chili varieties grown under cadmium stress. BMC Plant Biol.

[6] Dawar K, Asif M, Irfan M, Mian IA, Khan B, Gul N (2023). Evaluating the efficacy of activated carbon in minimizing the risk of heavy metals contamination in spinach for safe consumption. ACS Omega.

[7] Hossain ME, Shahrukh S, Hossain SA. Chemical Fertilizers and Pesticides: Impacts on Soil Degradation, Groundwater, and Human Health in Bangladesh. In: Environmental Degradation: Challenges and Strategies for Mitigation. Cham, Switzerland: Springer; 2022. p. 63–92.

[8] Isinkaralar K, Koc I, Erdem R, Sevik H (2022). Atmospheric Cd, Cr, and Zn deposition in several landscape plants in Mersin. Türkiye. Water Air Soil Pollut..

[9] Alhaji Adamu Y, Olaleye AA (2022). Speciation, mobility and potential toxicity of metals (Cr Co, Cu and Mn) in soil samples from dumpsites in kano metropolis. FUDMA J Sci.

[10] Gao H, Yang X, Wang N, Sun M, Xiao Y, Peng F (2022). Effects of different carbon types on the growth and chromium accumulation of peach trees under chromium stress. Agronomy.

[11] Huang S, Huang P, Hareem M, Tahzeeb-ul-Hassan M, Younis U, Dawar K (2024). Evaluating the hidden potential of deashed biochar in mitigating salinity stress for cultivation of fenugreek. Sci Rep.

[12] Anwar T, Shehzadi A, Qureshi H, Shah MN, Danish S, Salmen SH (2023). Alleviation of cadmium and drought stress in wheat by improving growth and chlorophyll contents amended with GA3 enriched deashed biochar. Sci Rep.

[13] Huang S, Huang P, Masood S, Iqbal MM, Naz T, Danish S (2024). Enhancing maize growth through the synergistic impact of potassium enrich biochar and spermidine. BMC Plant Biol.

[14] Shah SH, Hussain MB, Haider G, Haq TU, Zahir ZA, Danish S (2023). Acidified manure and nitrogen-enriched biochar showed short-term agronomic benefits on cotton–wheat cropping systems under alkaline arid field conditions. Sci Rep.

[15] Sarwar G, Anwar T, Malik M, Rehman H ur, Danish S, Alahmadi TA, et al. Evaluation of potassium-enriched biochar and GA3 effectiveness for Improving wheat growth under drought stress. BMC Plant Biol. 2023;23:615. 10.1186/s12870-023-04613-0 PMC10696804 38049735

[16] Ashraf F, Chen Y. Synergistic effects of biochar and arbuscular mycorrhizal fungi on enhancing Elymus elymoides growth in saline coastal soil. Pakistan J Bot. 2023;55 SI:119–26.

[17] Ibrahim MEH, Ali AYA, Elsiddig AMI, Zhou G, Nimir NEA, Agbna GHD (2021). Mitigation effect of biochar on sorghum seedling growth under salinity stress. Pakistan J Bot.

[18] Li X, Huang Y, Liang X, Huang L, Wei L, Zheng X (2022). Characterization of biochars from woody agricultural wastes and sorption behavior comparison of cadmium and atrazine. Biochar.

[19] Ramzan M, Jamshaid T, Ali L, Dawar K, Saba R, Jamshaid U (2023). Modulation of sunflower growth via regulation of antioxidants, oil content and gas exchange by arbuscular mycorrhizal fungi and quantum dot biochar under chromium stress. BMC Plant Biol.

[20] Alotaibi MO, Ikram M, Alotaibi NM, Hussain GS, Ghoneim AM, Younis U (2023). Examining the role of AMF-Biochar in the regulation of spinach growth attributes, nutrients concentrations, and antioxidant enzymes in mitigating drought stress. Plant Stress.

[21] Adhikari S, Timms W, Mahmud MAP (2022). Optimising water holding capacity and hydrophobicity of biochar for soil amendment – A review. Sci Total Environ.

[22] Sattar A, Ul-Allah S, Ijaz M, Sher A, Butt M, Abbas T (2022). Exogenous application of strigolactone alleviates drought stress in maize seedlings by regulating the physiological and antioxidants defense mechanisms. Cereal Res Commun.

[23] Sedaghat M, Emam Y, Mokhtassi-Bidgoli A, Hazrati S, Lovisolo C, Visentin I (2021). The Potential of the Synthetic Strigolactone Analogue GR24 for the Maintenance of Photosynthesis and Yield in Winter Wheat under Drought: Investigations on the Mechanisms of Action and Delivery Modes. Plants.

[24] Luqman M, Shahbaz M, Maqsood MF, Farhat F, Zulfiqar U, Siddiqui MH, et al. Effect of strigolactone on growth, photosynthetic efficiency, antioxidant activity, and osmolytes accumulation in different maize (*Zea mays *L.) hybrids grown under drought stress. Plant Signal Behav. 2023;18:2262795. 10.1080/15592324.2023.2262795 PMC10730227 37767863

[25] Tanwar R, Panghal A, Chaudhary G, Kumari A, Chhikara N. Nutritional, Phytochemical and Functional Potential of Sorghum: A Review. Food Chem Adv. 2023;3:100501.

[26] Nishio T, Nishio T, Kitashiba H (2017). Economic and Academic Importance of Radish. The radish genome.

[27] El-Beltagi HS, Maraei RW, Shalaby TA, Aly AA. Metabolites, Nutritional Quality and Antioxidant Activity of Red Radish Roots Affected by Gamma Rays. Agronomy. 2022;12.

[28] Mukherjee S, Chatterjee N, Sircar A, Maikap S, Singh A, Acharyya S (2023). A comparative analysis of heavy metal effects on medicinal plants. Appl Biochem Biotechnol.

[29] Cwielag-Piasecka I, Jamroz E, Medy nska-Juraszek A, Bednik M, Kosyk B, Polláková N. Deashed Wheat-Straw Biochar as a Potential Superabsorbent for Pesticides. Materials. 2023; 16, 2185. 10.3390/ma16062185 PMC10056329 36984065

[30] Ahmad I, Akhtar MJ, Zahir ZA, Naveed M, Mitter B, Sessitsch A (2014). Cadmium-tolerant bacteria induce metal stress tolerance in cereals. Environ Sci Pollut Res.

[31] Petersen RG, Calvin LD. Sampling. In: Klute A, editor. Methods of Soil Analysis: Part 1 Physical and Mineralaogical Methods, 5.1. 2nd edition. Madison, WI, USA: John Wiley & Sons, Inc., American Society of Agronomy, Inc. and Soil Science Society of America, Inc.; 1986. p. 33–51.

[32] Page AL, Miller RH, Keeny DR. Soil pH and lime requirement. In: Methods of Soil Analysis. 2nd edition. Madison: American Society of Agronomy; 1982. p. 199–208.

[33] Rhoades JD. Salinity: electrical conductivity and total dissolved solids. In: Sparks DL, Page AL, Helmke PA, Loeppert RH, Soltanpour PN, Tabatabai MA, et al., editors. Methods of Soil Analysis, Part 3, Chemical Methods. Madison, WI, USA: Soil Science Society of America; 1996. p. 417–35.

[34] Nelson DW a, Sommers L. Total carbon, organic carbon, and organic matter. Methods Soil Anal Part 2 Chem Microbiol Prop. 1983;9:539–79.

[35] Bremner M. Nitrogen-total. In: Sumner DL, Sparks AL, Page PA, Helmke RH, Loeppert NP, Soltanpour AM, et al., editors. Methods of Soil Analysis Part 3. Chemical Methods-SSSA Book Series 5. Madison, WI, USA: John Wiley & Sons, Inc.; 1996. p. 1085–121.

[36] Kuo S. Phosphorus. In: Sparks DL, Page AL, Helmke PA, Loeppert RH, Soltanpour PN, Tabatabai MA, et al., editors. Methods of Soil Analysis Part 3: Chemical Methods. SSSA, Madison, Wisconsin: John Wiley & Sons, Ltd; 2018. p. 869–919.

[37] Pratt PF. Potassium. In: Norman AG, editor. Methods of Soil Analysis, Part 2: Chemical and Microbiological Properties. Madison, WI, USA: John Wiley & Sons, Ltd; 2016. p. 1022–30.

[38] Gee GW, Bauder JW. Particle-size Analysis. In: Klute A, editor. Methods of soil analysis. Part 1. Physical and mineralogical methods. 2nd edition. Madison, WI, USA: John Wiley & Sons, Inc.; 2018. p. 383–411.

[39] Sardar R, Zulfiqar A, Ahmad S, Ali Shah A, Khalid Iqbal R, Hussain S, et al. Proteomic Changes in Various Plant Tissues Associated with Chromium Stress in Sunflower. Saudi J Biol Sci. 2021;:10.1016/j.sjbs.2021.12.042. 10.1016/j.sjbs.2021.12.042 PMC9072932 35531205

[40] Danish S, Kiran S, Fahad S, Ahmad N, Ali MA, Tahir FA (2019). Alleviation of chromium toxicity in maize by Fe fortification and chromium tolerant ACC deaminase producing plant growth promoting rhizobacteria. Ecotoxicol Environ Saf.

[41] European Union. Heavy Metals in Wastes, European Commission on Environment. 2002;:http://ec.europa.eu/environment/waste/studies/pd.

[42] Weatherley P. Studies in the water relations of the cotton plant. I. The field measurement of water deficits in leaves. New Phytol. 1950;49:81–97.

[43] Arnon DI (1949). Copper Enzymes in Isolated Chloroplasts Polyphenoloxidase in Beta vulgaris. Plant Physiol.

[44] Durak I, Yurtarslanl Z, Canbolat O, Akyol Ö (1993). A methodological approach to superoxide dismutase (SOD) activity assay based on inhibition of nitroblue tetrazolium (NBT) reduction. Clin Chim Acta.

[45] Cakmak I, Strbac D, Marschner H (1993). Activities of hydrogen peroxide-scavenging enzymes in germinating wheat seeds. J Exp Bot.

[46] Aebi H. Catalase in vitro. Methods Enzymol. 1984;105:121–6. 10.1016/s0076-6879(84)05016-3 6727660

[47] Nakano Y, Asada K (1981). Hydrogen peroxide is scavenged by ascorbate-specific peroxidase in spinach chloroplasts. Plant Cell Physiol.

[48] Hernández JA, Almansa MS (2002). Short-term effects of salt stress on antioxidant systems and leaf water relations of pea leaves. Physiol Plant.

[49] Jiang M, Zhang J (2001). Effect of abscisic acid on active oxygen species, antioxidative defence system and oxidative damage in leaves of maize seedlings. Plant Cell Physiol.

[50] Anderson ME. Determination of glutathione and glutathione disulfide in biological samples. Methods Enzymol. 1985;113:548–55. 10.1016/s0076-6879(85)13073-9 4088074

[51] Hodges DM, Andrews CJ, Johnson DA, Hamilton RI (1996). Antioxidant compound responses to chilling stress in differentially sensitive inbred maize lines. Physiol Plant.

[52] Lutts S, Kinet JM, Bouharmont J (1996). NaCl-induced senescence in leaves of rice (Oryza sativa L.) cultivars differing in salinity resistance. Ann Bot.

[53] Bates LS, Waldren RP, Teare ID (1973). Rapid determination of free proline for water-stress studies. Plant Soil.

[54] Steel RG, Torrie JH, Dickey DA (1997). Principles and Procedures of Statistics: A Biometrical Approach.

[55] OriginLab Corporation. OriginPro. Northampton.: OriginLab; 2021.

[56] Wu F, Gao Y, Yang W, Sui N, Zhu J (2022). Biological functions of strigolactones and their crosstalk with other phytohormones. Front Plant Sci.

[57] Kaniganti S, Bhattacharya J, Petla BP, Reddy PS (2022). Strigolactone, a neglected plant hormone, with a great potential for crop improvement: Crosstalk with other plant hormones. Environ Exp Bot.

[58] Shahid Z ul, Ali M, Shahzad K, Danish S, Alharbi SA, Ansari MJ. Author Correction: Enhancing maize productivity by mitigating alkaline soil challenges through acidified biochar and wastewater irrigation. Sci Rep. 2023;13:22544. 10.1038/s41598-023-49412-7 PMC10728204 38110477

[59] Sheikh L, Younis U, Shahzad AS, Hareem M, Noor Elahi N, Danish S. Evaluating the effects of cadmium under saline conditions on leafy vegetables by using acidified biochar. Pakistan J Bot. 2023;55 SI:33–9.

[60] Rahi AA, Hussain S, Hussain B, Baig KS, Tahir MS, Hussain GS (2022). Alleviation of Cd stress in maize by compost mixed biochar. J King Saud Univ - Sci.

[61] Wani KI, Zehra A, Choudhary S, Naeem M, Khan MMA, Khan R (2023). Exogenous strigolactone (GR24) positively regulates growth, photosynthesis, and improves glandular trichome attributes for enhanced artemisinin production in Artemisia annua. J Plant Growth Regul.

[62] Dawar K, Khan A, Mian IA, Khan B, Ali S, Ahmad S (2022). Maize productivity and soil nutrients variations by the application of vermicompost and biochar. PLoS One.

[63] Ahmad Rahi A, Younis U, Ahmed N, Arif Ali M, Fahad S, Sultan H, et al. Toxicity of Cadmium and Nickel in the Context of Applied Activated Carbon Biochar for Improvement in Soil Fertility. Saudi J Biol Sci. 2021;:10.1016/j.sjbs.2021.09.035. 10.1016/j.sjbs.2021.09.035 PMC8847926 35197740

[64] Iqbal J, Kiran S, Hussain S, Iqbal RK, Ghafoor U, Younis U (2021). Acidified Biochar Confers Improvement in Quality and Yield Attributes of Sufaid Chaunsa Mango in Saline Soil. Horticulturae.

[65] Manzoor S, Habib-ur-Rahman M, Haider G, Ghafoor I, Ahmad S, Afzal M, et al. Biochar and slow-releasing nitrogen fertilizers improved growth, nitrogen use, yield, and fiber quality of cotton under arid climatic conditions. Environ Sci Pollut Res. 2022;29:13742–55. 10.1007/s11356-021-16576-6 PMC8803770 34595718

[66] Das PP, Singh KR, Nagpure G, Mansoori A, Singh RP, Ghazi IA (2022). Plant-soil-microbes: a tripartite interaction for nutrient acquisition and better plant growth for sustainable agricultural practices. Environ Res.

[67] Prichystalova J, Holatko J, Hammerschmiedt T, Datta R, Meena RS, Sudoma M, Datta R, Meena RS (2021). Biochar Role in Soil Carbon Stabilization and Crop Productivity. Soil Carbon Stabilization to Mitigate Climate Change.

[68] Raja V, Qadir SU, Kumar N, Alsahli AA, Rinklebe J, Ahmad P (2023). Melatonin and strigolactone mitigate chromium toxicity through modulation of ascorbate-glutathione pathway and gene expression in tomato. Plant Physiol Biochem.

[69] Xiang L, Harindintwali JD, Wang F, Redmile-Gordon M, Chang SX, Fu Y, et al. Integrating biochar, bacteria, and plants for sustainable remediation of soils contaminated with organic pollutants. Environ Sci\& Technol. 2022;56:16546–66. 10.1021/acs.est.2c02976 PMC9730858 36301703

[70] Lingwan M, Pradhan AA, Kushwaha AK, Dar MA, Bhagavatula L, Datta S (2023). Photoprotective role of plant secondary metabolites: Biosynthesis, photoregulation, and prospects of metabolic engineering for enhanced protection under excessive light. Environ Exp Bot.

[71] Salam A, Khan AR, Liu L, Yang S, Azhar W, Ulhassan Z (2022). Seed priming with zinc oxide nanoparticles downplayed ultrastructural damage and improved photosynthetic apparatus in maize under cobalt stress. J Hazard Mater.

[72] Zhang B, Du H, Yang S, Wu X, Liu W, Guo J, et al. Physiological and Transcriptomic Analyses of the Effects of Exogenous Lauric Acid on Drought Resistance in Peach (*Prunus persica* (L.) Batsch). Plants. 2023;12:1492. 10.3390/plants12071492 PMC10097042 37050118

