# Supplementary material for: Sustainable remediation of chromium-contaminated soils: boosting radish growth with deashed biochar and strigolactone
Source: BMC Plant Biol. 2024 Feb 16;24:115. doi: 10.1186/s12870-024-04791-5 (PMC10870680; doi:10.1186/s12870-024-04791-5)
Supplement: Supplementary file 1 — Figure S1. Effect of treatments on the growth of Radish under normal condition and Cr toxicity (200 mg Cr/kg soil). [file 12870_2024_4791_MOESM1_ESM.docx]

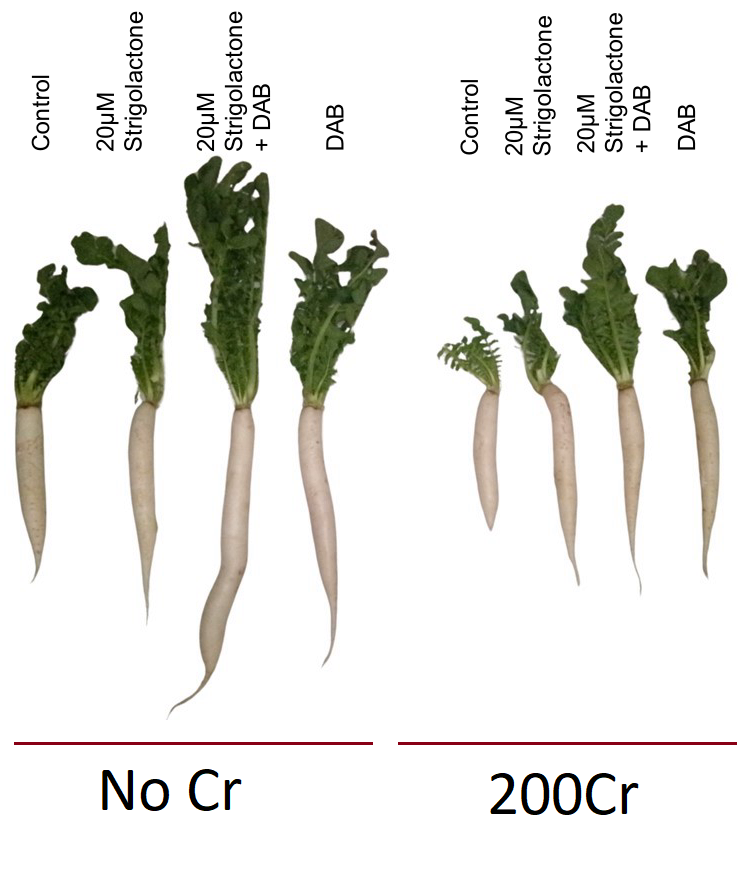


**Figure S1.** Effect of treatments on the growth o Radish under normal condition and Cr toxicity (200 mg Cr/kg soil).
